# Tools and Techniques to Accelerate Crop Breeding

**DOI:** 10.3390/plants13111520

**Published:** 2024-05-31

**Authors:** Krystal Williams, Mayavan Subramani, Lily W. Lofton, Miranda Penney, Antonette Todd, Gulnihal Ozbay

**Affiliations:** 1 Molecular Genetics and Epigenomics Laboratory, Department of Agriculture and Natural Resources, College of Agriculture, Science, and Technology, Delaware State University, Dover, DE 19901, USA; kdwilliams18@students.desu.edu; 2 Department of Plant Pathology, University of Georgia, Athens, GA 30602, USA; lily.lofton@uga.edu; 3 Toxicology & Mycotoxin Research Unit, US National Poultry Research Center, USDA-ARS, Athens, GA 30602, USA; 4 Plant Breeding and Genetics Section, School of Integrative Plant Science, Cornell University, Ithaca, NY 14853, USA; mlp254@cornell.edu; 5 One Health Laboratory, Department of Agriculture and Natural Resources, College of Agriculture, Science, and Technology, Delaware State University, Dover, DE 19901, USA

**Keywords:** speed breeding, CRISPR-Cas9, microbes, genomic selection, epigenomics, crop improvement

## Abstract

As climate changes and a growing global population continue to escalate the need for greater production capabilities of food crops, technological advances in agricultural and crop research will remain a necessity. While great advances in crop improvement over the past century have contributed to massive increases in yield, classic breeding schemes lack the rate of genetic gain needed to meet future demands. In the past decade, new breeding techniques and tools have been developed to aid in crop improvement. One such advancement is the use of speed breeding. Speed breeding is known as the application of methods that significantly reduce the time between crop generations, thereby streamlining breeding and research efforts. These rapid-generation advancement tactics help to accelerate the pace of crop improvement efforts to sustain food security and meet the food, feed, and fiber demands of the world’s growing population. Speed breeding may be achieved through a variety of techniques, including environmental optimization, genomic selection, CRISPR-Cas9 technology, and epigenomic tools. This review aims to discuss these prominent advances in crop breeding technologies and techniques that have the potential to greatly improve plant breeders’ ability to rapidly produce vital cultivars.

## 1. Introduction

The world population is estimated to increase to 10 billion by 2050 [1]. As the ratio of food producers to food consumers continues to decrease significantly, there is a rise in demand for greater quantities of food crops produced on less acreage and with less labor. This cannot be achieved solely with current crop varieties and agriculture practices [2]. The current crop productivity is not sufficient and must be doubled to meet the demand for plant-based products in 2050. Loss of diversity in modern crops due to bottlenecks of domestication, the time required to release new elite varieties, and suboptimal management of agricultural practices necessitate new technologies and approaches to enhance the rapid breeding of crops [2,3].

At its base, the term crop breeding refers to human efforts to enhance crop productivity, which involves the selection of desirable traits of plant variants to develop more efficient crops for production [4]. This process is driven by exploiting genetic variability within a species and is most often accomplished through techniques such as artificial pollination, embryo culture, chromosome doubling, and hybridization [5]. Yield, quality, hardiness, pest resistance, abiotic stress tolerance, and ease of processing are common traits that crop producers aim to improve with breeding [4] since these factors make up most of the connection between crop success and food availability. Despite their prior use in accelerating crop production, many existing crop breeding practices have been proven inefficient because of climate change and population growth. Additionally, even though its definitive effects on society are difficult to predict, climate change is still believed to pose a major threat to food security [6]. It has been predicted that under a regular population growth pathway, 120 million more people will be undernourished by the year 2080 [7,8], and this is due in part to food shortages stemming from consecutive seasons of unsuccessful crop growth.

**Figure 1:**
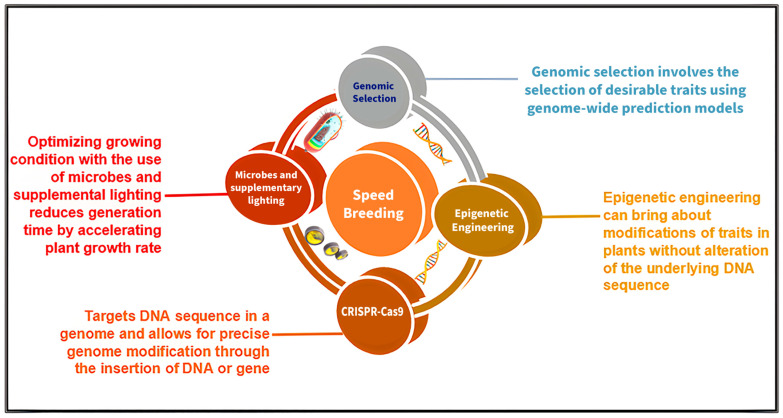
Plant breeding tools and techniques that can be utilized in speed breeding programs for rapid crop improvement.

Speed breeding is a method that shortens the breeding cycle, which permits rapid generation advancement, thereby accelerating crop research [9,10]. Speed breeding may be conducted in numerous ways, which include the extension of plants’ daily exposure to light and early seed harvest to reduce generation times for day-neutral crops [10]. Speed breeding, coupled with other breeding tools and technologies, may prove beneficial and effective in crop improvement (Figure 1). In this review, we discuss different tools and techniques for speed breeding. Each section includes a brief introduction about the tools and techniques and their application in speed breeding for accelerated crop improvement. The ability to access data from other research through scientific databases is imperative in the study of speed breeding because it aids in the expansion of both frequency of use and the purposes for which these techniques are employed. Additionally, since research databases have the potential to increase cost efficiency by allowing scientists to avoid costly mistakes and overall contribute to the creation of value within the scientific community [11], this review will also provide examples of studies in which the technologies discussed have already been implemented in crop breeding.

## 2. Optimization of Growing Conditions

Many crops, when grown under natural conditions, are limited to only one to two generations per year. This includes the use of winter nurseries, which advance breeding by allowing annual crops to produce additional generations per year [12]. This results in the need for several years to develop new cultivars of crops. To reduce the generation time, plants can be grown under optimal conditions to speed up growth rate and undergo physiological stress to flower early [10,13]. The use of artificial lighting and microbes are two methods that can be applied to speed up this process, thereby reducing the time it takes to develop new stable lines that can be evaluated agronomically.

### 2.1. Supplementary Lighting

**Figure 2:**
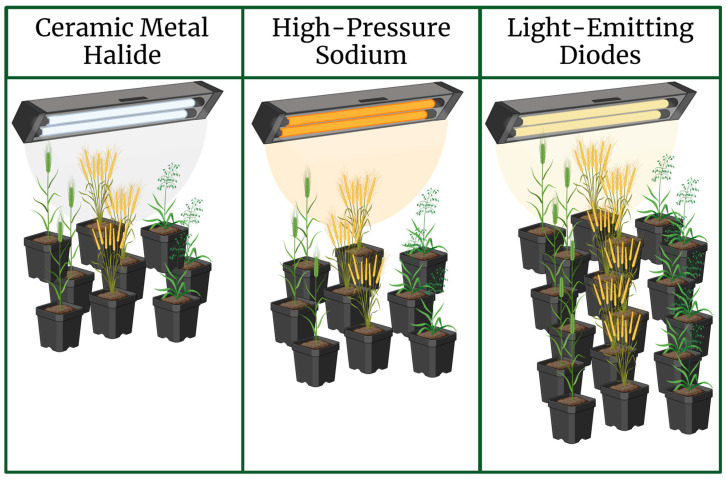
Effects comparison of supplementary lighting types on generation turnover. Aside from their overall energy efficiency and operating costs, light-emitting diodes (LEDs) outperform ceramic metal halide and high-pressure sodium lights in generations per year of barley, wheat, and oat crops [19]. Created with BioRender.com.

Plant growth and development are dependent on several photoreceptor families, which help plants respond accurately to extracellular environmental factors such as light [14]. Light impacts the molecular mechanism of plant development. Changes in gene expression profiles in response to light were reported in *Arabidopsis* and rice [15]. The plant responses to light vary depending on the wavelengths. As light is a necessary energy source for plant life, proper balancing of light energy enhances plant development overall. Light can be provided to plants naturally from the sun or with the use of supplementary sources. The use of supplementary lighting under control conditions is essential for growing healthy plants year-round. One of the greatest advantages to this is that it prevents the effects of seasonal variations in solar radiation and provides the plants with their required daily light integral (DLI) [16]. There are many forms of supplementary lighting, such as high-pressure sodium (HPS), light-emitting diodes (LEDs), ceramic metal halide, and fluorescent fixtures [17,18]. However, over the years, LED lamps have become the light source of choice due to the increase in light efficacy, low cost, and widespread availability. Recently, Stefański et al. [19] conducted a study comparing different types of supplemental lighting in cereal grain production and concluded that the use of LED lamps was better for energy efficiency over HPS and high-intensity discharge (HID) lamps in the breeding of oat (*Avena sativa* L.), barley (*Hordeum vulgare* L.), and wheat (*Triticum aestivum* L.) crops (Figure 2).

Developments in LEDs and their efficiency in the growth and development of crops [20] have opened new possibilities for efficient crop farming and plant growth in a controlled environment [21,22]. Metabolism, yield, and quality of the crop can be enhanced by the LEDs’ light quantity and quality [23]. LED fixtures normally require less electric power. They also maximize photon capture efficiency, which is essential in increasing the plant growth rate, thereby increasing generation turnover in some plants [12] (Figure 2).

Indoor cultivation methods are frequently used by plant breeders to speed up breeding to avoid adverse seasonal variations experienced outdoors [23]. Using LEDs in indoor cultivation reduces the overall production cost due to their high efficiency, low maintenance, and long-lasting qualities [24]. The application of LEDs is extended to the controlled environment agriculture (CEA) system, which includes greenhouses, climate rooms, and vertical farming [25].

Speed breeding can be applied independently of germplasm and accelerate generation time with early seed harvest in a controlled environment using optimal light intensity and light quality from LEDs. This has been successively achieved in wheat, barley, chickpea, pea, canola [26,27], and a few orphan crops [28]. For example, a speed breeding system employing an extended photoperiod of 22 h per day resulted in as many as six generations of spring wheat and spring barley per year [27], whereas speed breeding combined with speed vernalization systems resulted in up to five generations of winter barley and winter wheat [29]. LEDs can also be used in horticulture and floriculture to improve crop yield, pre- and post-harvest quality, phytochemicals, and nutritional contents [25]. Speed breeding in short-day crops such as soybean, rice, and amaranth enabled the growth of several generations in a year using crop-specific LED light adjustment in quantity and quality to speed up the flowering time and maturity. Thus, these smart lighting systems, such as crop-specific light regimes, will play a major role in enhancing urban farm production to meet food security challenges in growing urban populations by providing fast access to fresh food [30]. As part of the Bill and Melinda Gates Foundation, Lee Hickey and his research team, in partnership with the “International Crops Research Institute for the Semi-Arid Tropics (ICRISAT)”, assessed speed breeding short-day crops such as millet, sorghum, and pigeon pea by optimizing the methods to achieve early flower formation and rapid generation. Speed breeding through supplemental lighting in controlled environments has the potential to be integrated into high-throughput genotyping, genomic selection, and rapid generation of improved cultivars in breeding programs [2]. The fast growth and early flowering of plants lead to the possibility of producing a new generation in a shorter period, thereby facilitating the possibilities of mutant studies and the phenotyping of adult plants, hence speeding up the process of plant breeding.

### 2.2. Use of Microbes to Enhance Plant Growth

Another method of speed breeding for plant growth is the use of microbes. There are thousands of microbial species that form complex associations with plants. These associations can be beneficial for plant health [31]. Plants have the ability to shape their rhizosphere microbiome. This is based on the exudates they release from their roots. The different exudates can promote the growth of different microbial populations. Normally, plants of similar species have similar microbial populations [31]. The structural and functional characteristics of the roots contribute to the plant’s capacity to form relationships with these microbes, which affects the potential of the plant to acquire different nutrients [32]. The use of microbes for plant enhancement can reduce generation turnover. This is because some microbial species can regulate hormonal balance and produce plant growth regulators that increase the growth rate of plants [33]. Plant-growth-promoting rhizobacteria (PGPR) and mycorrhizae are examples of microbes that colonize and change the architecture of the roots of plants. PGPR produce phytohormones that enhance root branching and root hairs. This improves plant nutrition, thereby improving the entire physiology of the plant [34]. Mycorrhizal fungi, on the other hand, contribute towards solubilizing nutrients and helping with resistance against some plant pathogens [33]. These fungi have been used to alleviate disease in grain legumes. Hilou and his team demonstrated this in a research study they carried out. They utilized *Mosseae* spp. and *Rhizophagus irregularis* to show the bioprotective effects of mycorrhization against the plant pathogen *Aphanomyces euteiches*. This inoculation reduced the incidence of this root-rot-causing pathogen in legumes [35].

**Figure 3:**
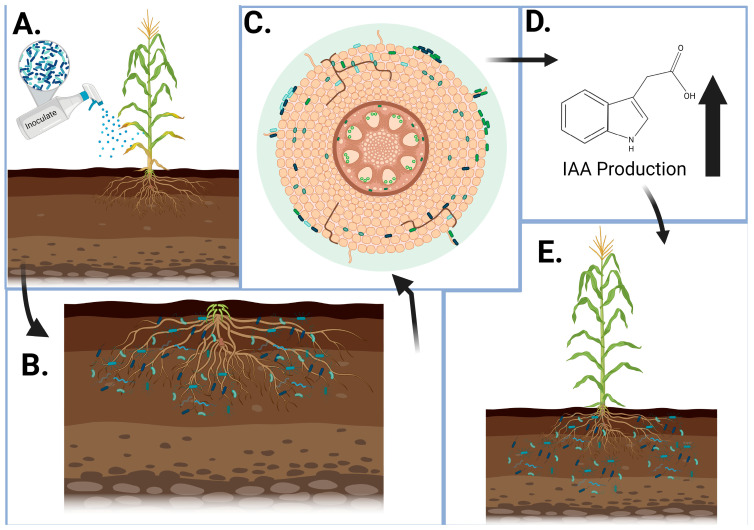
Bacterial inoculation of nutrient-deficient plants. (**A**) Soil growing a corn plant exhibiting nitrogen deficiency symptoms is spray-inoculated with PGP bacterial cocktail. (**B**) Bacterial colonies distribute throughout soil, then attach to and penetrate root tissue (**C**). This results in an increase in indole-3-acetic acid production within the plant (**D**), which supports plant growth mechanisms, targets the deficiency, and alleviates the symptoms (**E**) [39]. Created with BioRender.com.

Apart from fungi, bacteria also play an important role in providing protection against pathogens. For instance, the bacterial genus *Paenibacillus* has antagonistic activities against phytopathogens [36]. Also, rhizobia strains protect different legumes against root diseases such as damping off. *Rhizobium leguminosarum bv.* can be used to reduce the incidence of *Pythium* damping off in lentils and peas [37]. Endophytes, symbiotic microorganisms that live within plant tissue, have been reported to affect plant growth rates in many ways. Particularly, they have been shown to enhance the growth rate of *Pulicaria incisa* (Lam.) plants by triggering the production of indole acetic acid (IAA) [38], which is included in the main plant hormone class “auxin”, necessary for developmental processes in plants, including cell elongation, cell division, and initiation of responses to abiotic and biotic stimuli [39]. As highlighted earlier, beneficial microbes play an important role in growing healthy plants (Figure 3). This is crucial in reducing generational time in speed breeding.

## 3. CRISPR-Cas9

Many prokaryotes, including bacteria and archaebacteria, have evolved sophisticated immune systems encoded by clustered regularly interspaced short palindromic repeats (CRISPR) loci and CRISPR-associated (Cas) genes [40,41]. This CRISPR-Cas system creates an RNA-guided, adaptive immune system that provides immunity against bacteriophage infection and plasmid transfer [41]. To illustrate this, following infection from a bacteriophage, fragments of viral DNA are integrated as new spacers into the CRISPR array (short, identical repeats separated by unique spacers) of the host’s chromosome. This integration of foreign DNA provides a genetic record, or memory signature, of past infections, allowing the host to protect against future infections from the same agent [41,42]. Subsequent transcription and enzymatic processing of the CRISPR array yield short, mature CRISPR RNAs (crRNAs). The crRNA contains the new CRISPR spacer—an RNA sequence complementary to the foreign/invading genetic element—at the 5′ end and a piece of the CRISPR repeat sequence at the 3′ end [41]. The CRISPR-Cas immune system involves the transcription of CRISPR loci and accompanying Cas genes that encode proteins with predicted nuclease and helicase enzyme domains [42], which then use crRNA guidance to target and silence foreign RNA and DNA [40,43]. Upon a second infection, crRNAs combine with Cas proteins, forming an effector complex [44]. The crRNA spacer within the complex then hybridizes with the complementary, foreign target sequence, known as the protospacer (e.g., viral DNA or plasmids), allowing the complex to recognize the invasive genetic element [41,44]. This hybridization triggers sequence-specific cleavage and destruction of invading genetic elements by Cas nucleases [41], which prevents the propagation of foreign nucleic acids.

### 3.1. CRISPR-Cas9 Technology

**Figure 4:**
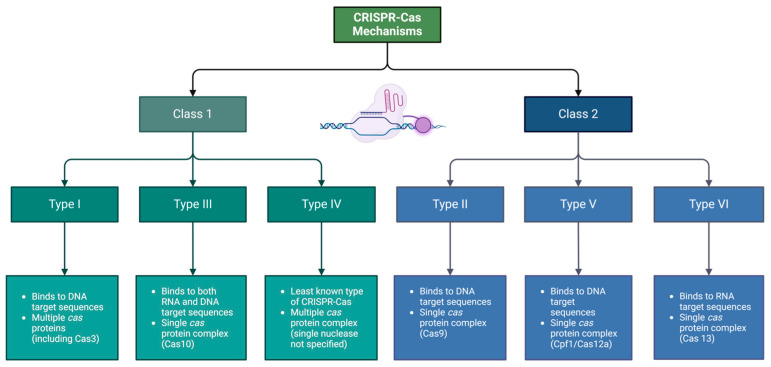
Differentiation of CRISPR-Cas mechanisms. CRISPR-Cas mechanisms share similar functions but differ in structure and/or methodology. The mechanisms are divided into two classes: class 1, which indicates that the mechanisms have more complex, multi-subunit crRNA effector complexes, and class 2, which consists of mechanisms that employ less complex, single-subunit crRNA effector complexes [45]. Created with BioRender.com.

CRISPR-Cas systems have been grouped into six different types (I–VI) across two classes, which each use different molecular mechanisms—Cas proteins and crRNAs—to recognize and cleave invading genetic materials [41]. CRISPR mechanisms are separated into broad classes based on the complexity of their crRNA effector complexes (class 1: multi-subunit crRNA effector complex; class 2: single crRNA effector complex) [45]. Types I, II, and V use crRNAs that bind to DNA target sequences, while types III and VI use crRNAs that pair with RNA target sequences [46]. System types I and III use a large complex of multiple Cas proteins to achieve crRNA-guided targeting, binding, and target sequence degradation [41,42]. In contrast, type II CRISPR systems use a single protein, the Cas9 DNA endonuclease, to recognize target sequences and cleave them at a site-specific location [41]. In particular, a short sequence motif located adjacent to the crRNA-targeted sequence, known as the protospacer-adjacent motif (PAM), fulfills an important function in nucleic acid targeting and degradation in most CRISPR-Cas systems, specifically the type II system [41,42]. In type II, the CRISPR-Cas9 system, a small noncoding RNA, called the trans-activating crRNA (tracrRNA), binds with the repeat sequence in the crRNA [41]. The crRNA is then bound to the protospacer and the tracrRNA, giving it a unique dual-RNA structure. Together, this duality acts as a guide to direct the Cas9 enzyme to cleave any DNA that contains the complementary target sequence (protospacer) and an adjacent PAM. By changing the spacer sequence within the crRNA (guide RNA), the CRISPR-Cas9 system is proficient in targeting almost any DNA sequence in a genome and creating a sequence-specific, double-stranded cut. This cleavage is then repaired randomly through indels occurring at the breakage site—or by directed repair through the intentional insertion of a homologous repair template [41]—allowing for precise genome modification through the insertion of DNA, may it be a gene of interest or other (Figure 4).

Since the early 2000s, the CRISPR-Cas9 system has been proven to be a substantial RNA-guided DNA targeting technology for genome editing, as well as a host of other applications such as targeted mutagenesis, genome imaging, epigenetic modulation, and transcriptional perturbation [41]. Simply put, the CRISPR-Cas9 technology has two main components that may be delivered as a single plasmid when used for genetic engineering. This includes (a) the bacterial Cas9 endonuclease protein and (b) a specifically designed single-guide RNA (sgRNA) containing the protospacer sequence that is homologous to the target DNA. Additionally, to better understand the efficiency of the CRISPR-Cas9 system and its binding abilities, there needs to be improved knowledge of the effect of chromatin accessibility as well as the epigenomic environment at the target site [47].

### 3.2. Applications of CRISPR-Cas9 Technology in Speed Breeding

This section focuses on the use of CRISPR-Cas9 technology for advanced crop and agricultural product improvement (for example, disease and pathogen resistance, increased shelf life, and browning resistance [47]) through genetic engineering and plant breeding. Genetic engineering, when combined with speed breeding methods, can hugely accelerate crop improvement and breeding.

In plants, the CRISPR-Cas9 technology can be delivered using multiple methods, including transient and transformation platforms, such as protoplast transfection, agroinfiltration, and particle bombardment of callus tissue. Many initial groups demonstrated that CRISPR-Cas9 technology could successfully transform model species—such as *Arabidopsis* and tobacco—as well as major crops, including rice, wheat, sorghum, maize, tomato, potato, and soybean—all of which have been previously summarized [47]. Although successful, the gene editing process is time-consuming, requiring tissue-culturing and specialized labs with physical containment spaces available for the CRISPR-Cas9 system delivery and action [26]. However, innovative systems, such as “ExpressEdit” (originally described by Hickey et al. [26]), incorporate rapid gene editing directly into the speed breeding program, reducing the time restraint of traditional gene editing by removing the in vitro manipulation. ExpressEdit is not yet a routine practice, but it has huge implications for the future of speed breeding and crop improvement. This approach involves applying both the Cas9 gene and the produced sgRNA sequence directly to plants. Together, for example, this sgRNA could confer a trait for disease resistance. Progeny of the exposed plants could be screened for the new trait, and those expressing the trait and without Cas9 could be selected [26]. These plants would then be indistinguishable from those that obtained the trait through natural mutation and would be carried through the traditional breeding steps of field evaluation and variety release while optimally avoiding the genetically modified organism label. Alternately, Cas9 could solely remain in some plants—then becoming “CRISPR-ready” plants—that would be primed for more cycles of gene editing through the application of new sgRNA to target different traits [26].

Liu et al. recently conducted a study that used CRISPR-Cas9 to produce lines of hybrid wooly tomato seeds [48]. The key to generating successful hybrid seeds is to establish male-sterile lines that would maintain purity within the tomato lines. Previously, this could only be accomplished through time-consuming emasculation processes; however, the implementation of the CRISPR-Cas9 system into this process allowed for marker-assisted screening that initiated mutations, which facilitated male sterility in woolly tomato hybrid seeds [48].

As briefly discussed above, there are multiple ways to deliver the CRISPR-Cas9 system to plants for targeted genome editing. This is also true for tissue culture-free approaches. For example, embryos or protoplasts may be used as the target tissue. However, to avoid tissue-culturing, this rapid gene editing approach would ideally target mature seeds or germinating seedlings [26,28]. In subsequent generations, phenotyping would also allow for the stacking of traits [26,49]. Another method includes the use of clay nanosheets. There is potential for the engineering of clay sheets for use as a delivery method for both the Cas9 protein and, for example, an RNA interference (RNAi) construct for virus resistance [50]. Commonly, these components are delivered via viral vectors (e.g., geminiviruses) [51] or particle bombardment (a biological ballistic DNA delivery method) to the shoot apical meristems, which also allows for the editing of pollen and inflorescence tissue [26,49].

CRISPR-Cas technology has also been implemented with the goal of inducing synthetic apomixis in plants to improve hybrid vigor in crop breeding programs [52]. Apomixis is a somatic method of asexual reproduction that produces a clone identical to the mother plant in genetic makeup [53]. Recently, the induction of synthetic apomixis through colchicine-mediated mutagenesis has been studied in an Egyptian rice hybrid, resulting in the fixation of heterosis that would typically be broken by population segregation [53]. However, one of the more germane goals of developing apomixis traits in plant breeding lines is to facilitate the transgenerational expression of phenotypes conferred either through transgenic or traditional breeding methods. This is demonstrated by Liu et al. [54], who were able to achieve transgenerational expression of phenotypes in a rice hybrid through the induction of synthetic apomixis.

Traditional transformation methods with crop plants that already exhibit apomixis traits often display differences in the immediate generations but require multiple trials before transgenes are observable and functional in subsequent generations, as demonstrated by Aragão et al. [55]. However, this phenomenon has been shown to be reversed in instances utilizing more innovative transformation systems. For example, Feng et al. [56] investigated the heritability of CRISPR-Cas-induced gene transformations and found that, while some modifications are not heritable in the initial plants of interest, they are heritable in future generations.

Currently, several genes have been successfully cloned for crop improvement, many of them being resistance (R) genes to various biotic and abiotic stressors [57]. The *Helminothosporium carbonum* susceptibility 1 (Hm1) gene from maize, which encodes the detoxification of the fungal pathogen *Cochliobolus carbonum*, was the first R gene to be cloned [58] and paved the way for the rise in R gene cloning among crop breeders. Similarly, Luo et al. [59] conducted a pioneering study that led to the identification of a five-transgene stack capable of conferring genetically linked, broad-spectrum resistance against a wheat stem rust pathogen *Puccinia graminis* f. sp. *Tritici* in wheat. In abiotic stress R gene research, advanced QTL mapping of rice (*Oryza sativa* L.) populations resulted in the targeting of several genes related to salt tolerance, providing a foundation for salt tolerance gene cloning processes as well as molecular-assisted selection breeding in rice [60]. The improvement in plant immunity through the conferral of resistance genes aids and accelerates the breeding process by protecting crops from detrimental diseases and conditions in order to help preserve potential yield.

Gene editing using CRISPR-Cas9 technology coupled with speed breeding provides a powerful method to develop next-generation crop varieties capable of superior yield and stress resistance. Such crops have great promise for meeting the ever-rising demands of population growth.

## 4. Genomic Selection

With the continued advancement of field and laboratory techniques, such as LED applications and CRISPR/Cas9, the potential for in silico speed breeding schemes must also be considered. The leaps and bounds made in both high-density genome sequencing and computational bioinformatics technologies present plant breeders with powerful tools for rapid variety development. Genomic selection (GS), also called whole-genome selection or genomic prediction, represents such a tool.

### 4.1. Overview of Genomic Selection Methodology

The goal behind genomic selection is to identify desirable traits in individuals or lines of the target species without the need for lengthy field trials or phenotyping. This is conducted in two main phases: first is the training phase, in which a population of individuals is carefully selected based on their genetic closeness to the breeding populations as well as their ability to represent the potential genetic diversity of that population. These individuals are genotyped using hundreds of thousands of genome-wide markers and are phenotyped for the trait or traits of interest [41]. Combined, these collected data are used to broadly correlate genotype to phenotype using a generated prediction model. Two subsets of populations comprise this phase: the training population around which the model is formed, and the validation population, which is used to test the model’s accuracy [51]. This model is then used in the second phase: selection from the breeding population. At this stage, the GS model can provide genomic estimated breeding values (GEBVs) based on genomic data input and predicted marker effects. Breeding population lines need only be genotyped to select individuals for advancement in or removal from the breeding program [61,62].

### 4.2. Genomic Selection Method for Speed Breeding Programs

Genomic selection, which was first proposed by Meuwissen et al. [63], has been widely successful in dairy cattle breeding programs across the United States, given that this method allows breeders to estimate breeding values of sires without having to wait years for maturation and phenotyping of the progeny. The potential benefits of this breeding method in field crops such as corn, wheat, and rice have been evaluated in crop breeding research to a limited extent and have yet to be fully utilized [62]. Superficial similarities of GS to often-used techniques, such as marker-assisted selection (MAS), as well as initial cost prohibitions, have contributed to the slow uptake of this method by plant breeders. However, the advantages of this technique in a speed breeding program must be considered. 

Common molecular breeding techniques such as MAS rely on detailed knowledge of the biological function of the trait and trait genetics, leading to a breeding-by-design approach that requires years of research and validation before implementation. Genomic selection’s use of whole-genome prediction models removes that limitation, simultaneously accounting for groups of predictors rather than individual quantitative trait loci (QTLs) [64]. This allows breeding programs to select for traits not yet studied in detail. Additionally, MAS is limited in its ability to identify complex, multi-gene traits. This leaves many key economic traits, such as yield and tolerance traits, unattainable by MAS alone. Genomic selection models, on the other hand, are able to capture most minor effect loci that contribute to the target trait, so long as the trait loci and genome-wide markers are in some degree of linkage disequilibrium [65].

As previously mentioned, one of the most notable advantages of genomic selection is a significant decrease in the time needed to make large genetic gains. Field trials for accurate phenotyping of breeding populations require years of expensive testing in a variety of conditions and locations; this problem is overstepped by genomic selection in the model’s ability to predict desirable traits simply from observation of the genotype [66]. A prominent example in oil palm (*Elais guineensis*) demonstrated a reduction in the breeding program’s selection cycle from 19 to 6 years after the implementation of GS [64]. Time and funds needed for field trials of breeding populations can be significantly reduced, resulting in greater genetic gain per unit of time. As crop sequence databases continue to expand, the resources available to breeders allow for the implementation of genomic selection with greater accuracy and flexibility in statistical model design.

Though GS breeding designs have immense potential for expediting the breeding process, the efficacy of genomic selection and prediction in plant breeding can be hindered by the complexity of target genes and polygenic trait stacks of interest [67]. Full efficiency as a breeding tool requires accommodation for all potential setbacks, including heritability and complex general architecture. However, strides have been made in tackling this challenge. Recently, the cooking time of various common bean (*Phaseolus vulgaris* L.) populations, which is a highly complex trait that involves several genomic regions, was predicted via GS analysis methods [68]. Given the complexity of the cooking time trait, the implementation of GS methods allowed for expeditious analysis of the involved genomic pathways. While the application of GS in speed breeding programs is still limited due to little precedent in the plant breeding community and initial costs of sequencing, these hurdles will become increasingly crossable as more researchers explore the potential for this technique.

## 5. Epigenomics/Epigenetics

Epigenomics is referred to as the sum of all changes in gene expression and cellular function as a result of DNA and histone modifications and synthesis of non-coding RNA without altering the underlying DNA sequence [69]. DNA and histone modifications play key roles in the regulation of gene expression and, evidently, the development of plants and their responses to the environment [70]. DNA methylation, histone acetylation, ubiquitination, and phosphorylation are common examples of these modifications. DNA methylation is one of the most studied epigenetic marks in plants [71]. This type of modification, along with others, can be monitored and mapped by using high-throughput next-generation sequencing technologies, which may provide a deeper understanding of the role of histone and DNA modifications in their regulation of plant growth and development as well as their responses to biotic and abiotic stresses [72]. DNA methylation of cytosine occurs in the fifth position on the cytosine base by the enzyme DNA methyltransferase and occurs in three different signature nucleotide sequence contexts: CG, CHG, and CHH [73]. These patterns are inherited through cell division and may contribute to phenotypic variability.

**Figure 5:**
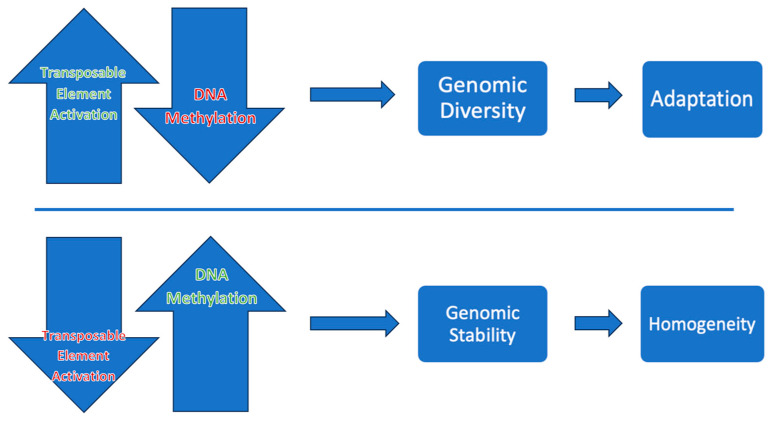
Epigenetic relationship between transposable element (TE) activity and DNA methylation. When DNA methylation is lower, TE activation is higher, facilitating genomic diversity. Suppression of TE activation encourages genomic stability [74].

DNA methylation patterns may be altered by environmental stimuli such as abiotic and biotic stressors or by underlying genetic factors [74]. Deciphering patterns of DNA methylation and genetic information on epigenetic marks can provide insights into how epialleles control genes and transcript expression during the plant’s developmental stages and its response to stress in different crop genotypes [72]. The CG pattern, also known as gene body methylation (gbM), refers to methylation that takes place within the transcriptional regions and is linked to mild expression levels. The pattern CG transposable element (TE)-like methylation is associated with the suppression of TE activities (Figure 5). TE activities are found to play important roles in increasing genomic diversity, making it applicable for breeding [33].

The relationship between DNA methylation and gene expression may be exploited by purposeful disturbance of DNA methylation by external interventions, which may help to fast-track variability for crop improvement. This can be achieved by using methods that are similar to those that are utilized in mutation breeding or the application of DNA methyltransferase inhibitors, which leads to genome-wide DNA demethylation. Targeted epigenome techniques that can alter DNA methylation can also be utilized, which may result in phenotypic changes as well as changes in gene expression [75].

A well-known method of epigenetic engineering exists in the form of RNA interference. RNAi is a method of post-transcriptional gene silencing facilitated by the expression of double-stranded RNA (dsRNA) [76]. Small molecules of interfering RNA are used to make a specific gene more expressive in its transcriptome, facilitating a certain trait [77]. Cleavage of dsRNA (i.e., microRNA and small interfering RNAs) into active small non-coding RNAs is facilitated by the Dicer (or Dicer-like) multidomain endoribonuclease [78]. These small non-coding RNAs, which are involved in the gene regulatory process, are incorporated into a second ribonuclease system—consisting of the RNA-induced silencing complex (RISC) as well as argonaute and other effector proteins—that initiates the gene silencing process through targeted degradation of messenger RNAs [78,79]. RNAi systems can be delivered transiently through classic transformation delivery systems, including particle bombardment, viral delivery, and *Agrobacterium*-mediated delivery [79].

### Application of Epigenetics in Breeding

Epigenetic engineering can bring about modifications of traits in plants without altering the underlying DNA sequence. Several epialleles have been discovered that give rise to phenotypic and morphological variation; this process, however, may take some time since it is dependent on spontaneous or natural epimutations. Epigenetic variations are known to affect important traits in crop plants; therefore, “manipulation of stably inherited epigenetic variation may be a powerful tool in plant breeding” [69]. Targeted DNA/chromatin-modifying enzymes can be used to epigenetically manipulate an epigenetic mark that influences gene expression. These modifying enzymes are known to successfully manipulate epigenetic marks at the target site. These enzymes are also used in the up- and down-regulation of gene transcription [69].

RNAi technology has been used to address several types and degrees of both biotic and abiotic stressors [80]. By activating abnormal expression of defensive genes against plant pathogens and other invading phenomena, RNAi has facilitated the conferral of traits including bacterial resistance [81], fungal resistance [82], insect resistance [83], drought tolerance [84,85], heat stress tolerance [86], and salinity tolerance [87]. RNAi allows for the assessment of gene function, the development of abiotic and biotic stress-resistant crops, the development of disease-resistant crops [76,77,88], and the process of plant metabolic engineering [78].

Understanding how epigenetic changes affect multiple copies of a gene may provide insight into the development of crops with tolerance to multiple stresses. To ensure that the crops will be capable of withstanding the changes to environmental conditions, there needs to be a stable inheritance of the adaptive epiallele. Once the inheritance of the DNA methylation is stable, then the epiallele will also be stable, thus reducing the formation of new epialleles. For example, Ketumile et al. [89] found that epigenetic variation in sorghum (*Sorghum bicolor*) has demonstrated the possibilities of breeding sorghum for crop resilience by focusing on the Mutator S HOMOLOG 1 (MSH1) gene. In this study, RNAi-induced silencing of the MSH1 gene in sorghum triggered intergenerational epigenomic changes, which is a phenomenon known as methylation repatterning [80,89,90]. If the DNA modification is not stable, it can increase the formation or loss of the epialleles within that generation. Therefore, the stability of the epigenetic mark over generations plays a crucial role in its ability to aid in crop improvement [69]. Two separate studies found that *Arabidopsis* exposed to salinity [91] and rice exposed to drought stress [92] for several generations displayed increased rates of changes in DNA methylation, which indicates that methylation stability may be altered by environmental conditions.

Plant breeding through epigenetic engineering may take several generations for plants to display phenotypic variability or inherit desired traits; therefore, utilizing speed breeding with epigenetic tools can shorten generation time while also producing crops that are tolerant to various stresses. Since epigenetic regulation of gene expression has an impact on crop traits, it can be used as a powerful tool in plant breeding. One important benefit is that it does not involve changes to the DNA sequence and is a natural pathway.

## 6. Discussion and Future Directions

**Figure 6:**
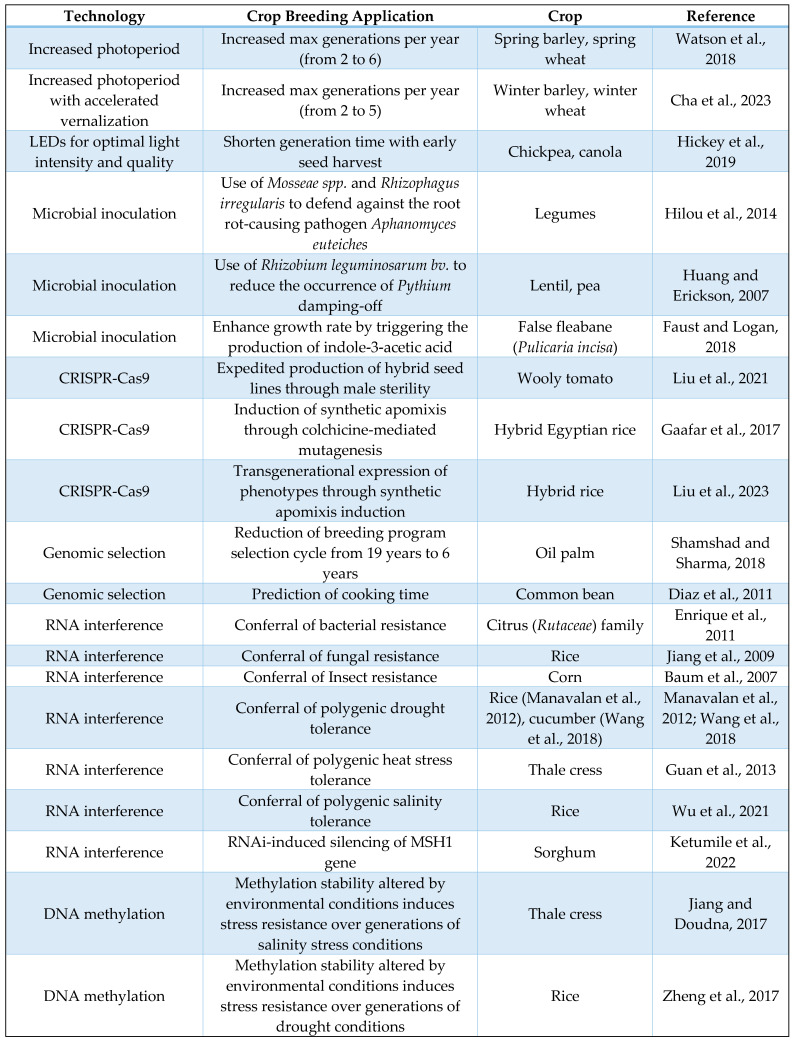
List of utilization examples of mentioned technologies [26,27,29,35,37,38,48,53,54,64,68,81,82,83,84,85,86,87,89,91,92].

For the development of speed breeding programs, up-to-date technologies will always be in high demand. The implementation of speed breeding technologies, such as those discussed above, has already become a necessity in a world of ever-increasing population and diminishing resources. Speed breeding techniques and technologies offer solutions to problems that have inhibited efficient crop growth for years. The discussed methods and examples (Figure 6) not only address the circumstances known to slow down the crop breeding and production processes but also repair aspects of cropping systems that fuel inefficiency overall. Speed breeding uses regulated environmental conditions such as temperature, light intensity, and daytime control, which results in a shortened generation time [93]. In implementing speed breeding techniques into commercial cropping systems, crop breeders will be able to mitigate food shortages due to changes in natural phenomena, which is one of the societally detrimental effects of climate change.

Speed breeding techniques and innovations have the potential to foster vast improvements within the crop breeding industry, but the advancement of these technologies comes with natural setbacks and challenges. For example, genotype-by-environment interactions in genomic selection trials complicate breeding efforts by devaluating the selection of superior individuals for breeding, given that these interactions affect generational yield stability. While the environmental effect has a major impact on interactions, the general selection process is more affected by the relationship between production environments and selection environments [94]. Classical selection approaches typically take place in ideal, sterile production environments, which often results in inadequate consideration of the possibility that more unpredictable aspects of environmental variability (i.e., climatic conditions) will impact plant breeding trials. Genotype-by-environment interactions are important for identifying the genetic basis from which plants adapt to new environments. [95]. Focusing on genotype by environmental interactions in crop plants enables and encourages breeders to select not just based on projected hybrid vigor but also based on the versatility of species or their ability to fill a specific niche in an ecosystem [96].

Availability and ease of use pose more challenges for the integration of speed breeding innovations. For example, while GS systems simplify the breeding process overall, there is a need for more standardized programs, including human resource development for genomic selection model creation/utilization, methods for advanced data collection, and recordkeeping [97]. This general lack of guidance makes it difficult for those wanting to execute genomic selection practices in their own research [98]. Conversely, since its popularity as a user-friendly genome editing solution is increasing, CRISPR-Cas9 is becoming more widely available for use in both academia and the private sector [52,99]. Similarly, RNAi technology has been proven to be highly applicable to crop speed breeding efforts and has been put into practice across the field of agriculture [100].

Developing and perfecting new technologies that advance the study of speed breeding can occur with many types of plants, but it is often easier to work toward developing a standard by testing new technologies on a well-studied species. It is for this reason that maize (*Zea mays* L.) is often used as a model plant for new cropping technologies. For example, Singh et al. [101] used maize to model speed breeding lighting and temperature conditions, finding that it could tolerate a higher light intensity than other plants, which allowed it to withstand the level of light associated with speed breeding conditions. Maize also has a high radiation use efficiency, which is identified as the micromoles of matter produced per mole of photosynthetically active photons absorbed by green canopy components [101]. Plus, the yield of maize per season is very heavily dependent on ideal climatic conditions, which are threatened by the onset of abiotic stresses [102]. Maize was used as the subject of a 16-line multiparent advanced generational intercross (MAGIC) to further identify the genetic basis of genotype by environment interactions. Sixteen maize lines were investigated across five temperate environments experiencing contrasting climatic conditions and a variety of management practices [95]. Maize’s general growth characteristics, susceptibility to environmental factors, and history as a model in plant science research overall [103,104] have solidified its place as a model crop for the testing and development of speed breeding technologies.

Future studies have the potential to pioneer the integration of key molecular techniques such as genomic selection, CRISPR-Cas9, and epigenetic engineering with the growing stores of data from all varieties of “omics” research, from transcriptomics to proteomics, to develop a network of systemic breeding approaches. Looking forward, the development of MiMe (mitosis instead of meiosis) genotyping technology has shown potential for success in future studies of its integrations within speed breeding systems [52]. Additional study of crop apomixis in mutants also paves the way for studies allowing the production of transgenerational trait expression in mutants, thereby improving the efficiency of clonal propagation in crop plants [52]. Combined with optimized growing conditions to shorten generational times, these technologies will continuously enhance crop breeders’ ability to rapidly produce high-quality and hardy cultivars capable of meeting the challenges of our growing world.

## References

[1] Lutz W., Kc S. (2010). Dimensions of global population projections: What do we know about future population trends and structures?. Philos. Trans. R. Soc. Lond. Ser. B Biol. Sci..

[2] Voss-Fels K.P., Stahl A., Hickey L.T. (2019). Q&A: Modern crop breeding for future food security. BMC Biol..

[3] Kulchin Y.N., Zmeeva V.N., Subbotin E.P., Kostyanko A.A. (2018). The effect of multispectral light emitting diodes (LEDs) on the activation of morphogenic processes in cell culture of rice *Oryza sativa*. Defect Diffus. Forum.

[4] Breseghello F., Coelho A.S.G. (2013). Traditional and modern plant breeding methods with examples in rice (*Oryza sativa* L.). J. Agric. Food Chem..

[5] Anand A., Subramanian M., Kar D. (2023). Breeding techniques to dispense higher genetic gains. Front. Plant Sci..

[6] Lenaerts B., Collard B.C.Y., Demont M. (2019). Review: Improving global food security through accelerated plant breeding. Plant Sci..

[7] Raj S., Roodbar S., Brinkley C., Wolfe D.W. (2022). Food Security and Climate Change: Differences in Impacts and Adaptation Strategies for Rural Communities in the Global South and North. Front. Sustain. Food Syst..

[8] Fischer G., Shah M., Tubiello F.N., van Velhuizen H. (2005). Socio-economic and climate change impacts on agriculture: An integrated assessment, 1990–2080. Philos. Trans. R. Soc. Lond. Ser. B Biol. Sci..

[9] Alahmad S., Dinglasan E., Leung K.M., Riaz A., Derbal N., Voss-Fels K.P., Able J.A., Bassi F.M., Christopher J., Hickey L.T. (2018). Speed breeding for multiple quantitative traits in durum wheat. Plant Methods.

[10] Poulet L., Massa G.D., Morrow R.C., Bourget C.M., Wheeler R.M., Mitchell C.A. (2014). Significant reduction in energy for plant-growth lighting in space using targeted LED lighting and spectral manipulation. Life Sci. Space Res..

[11] Figueiredo M.S.N., Pereira A.M. (2017). Managing Knowledge—The Importance of Databases in the Scientific Production. Procedia Manuf..

[12] Cook T.M., Isenegger D., Dutta S., Sahab S., Kay P., Aboobucker S.I., Biswas E., Heerschap S., Nikolau B.J., Dong L. (2023). Overcoming roadblocks for in vitro nurseries in plants: Induction of meiosis. Front. Plant Sci..

[13] Turck F., Fornara F., Coupland G. (2008). Regulation and Identity of Florigen: FLOWERING LOCUS T Moves Center Stage. Annu. Rev. Plant Biol..

[14] Chen M., Chory J., Fankhauser C. (2004). Light Signal Transduction in Higher Plants. Annu. Rev. Genet..

[15] Jiao Y., Ma L., Strickland E., Deng X.W. (2005). Conservation and divergence of light-regulated genome expression patterns during seedling development in rice and Arabidopsis. Plant Cell.

[16] Fang L., Ma Z., Wang Q., Nian H., Ma Q., Huang Q., Mu Y. (2021). Plant Growth and Photosynthetic Characteristics of Soybean Seedlings Under Different LED Lighting Quality Conditions. J. Plant Growth Regul..

[17] Ghosh S., Watson A., Gonzalez-Navarro O.E., Ramirez-Gonzalez R.H., Yanes L., Mendoza-Suárez M., Simmonds J., Wells R., Rayner T., Green P. (2018). Speed breeding in growth chambers and glasshouses for crop breeding and model plant research. Nat. Protoc..

[18] Nelson J.A., Bugbee B. (2014). Economic analysis of greenhouse lighting: Light emitting diodes vs. high intensity discharge fixtures. PLoS ONE.

[19] Stefański P., Siedlarz-Slowacka P., Matysik P., Rybka K. (2022). Efficiency of LED lamps used in cereal crop breeding greenhouses. Int. J. Agric. Biol. Eng..

[20] Morrow R.C. (2008). LED Lighting in Horticulture. HortScience Horts.

[21] Agarwal A., Gupta S.D. (2016). Impact of Light-Emitting Diodes (LEDs) and Its Potential on Plant Growth and Development in Controlled-Environment Plant Production System. Curr. Biotechnol..

[22] Danila E., Lucache D.D. Efficient lighting system for greenhouses. Proceedings of the 2016 International Conference and Exposition on Electrical and Power Engineering (EPE).

[23] Monostori I., Heilmann M., Kocsy G., Rakszegi M., Ahres M., Altenbach S.B., Szalai G., Pál M., Toldi D., Simon-Sarkadi L. (2018). LED lighting–modification of growth, metabolism, yield and flour composition in wheat by spectral quality and intensity. Front. Plant Sci..

[24] Singh D., Basu C., Meinhardt-Wollweber M., Roth B. (2015). LEDs for energy efficient greenhouse lighting. Renew. Sustain. Energy Rev..

[25] Bantis F., Smirnakou S., Ouzounis T., Koukounaras A., Ntagkas N., Radoglou K. (2018). Current status and recent achievements in the field of horticulture with the use of light-emitting diodes (LEDs). Sci. Hortic..

[26] Hickey L.T., Hafeez A.N., Robinson H., Jackson S.A., Leal-Bertioli S.C.M., Tester M., Gao C., Godwin I.D., Hayes B.J., Wulff B.B.H. (2019). Breeding crops to feed 10 billion. Nat. Biotechnol..

[27] Watson A., Ghosh S., Williams M.J., Cuddy W.S., Simmonds J., Rey M.D., Asyraf Md Hatta M., Hinchliffe A., Steed A., Reynolds D. (2018). Speed breeding is a powerful tool to accelerate crop research and breeding. Nat. Plants.

[28] Chiurugwi T., Kemp S., Powell W., Hickey L.T. (2019). Speed breeding orphan crops. Theor. Appl. Genet..

[29] Cha J.-K., Park H., Choi C., Kwon Y., Lee S.-M., Oh K.-W., Ko J.-M., Kwon S.-W., Lee J.-H. (2023). Acceleration of wheat breeding: Enhancing efficiency and practical application of the speed breeding system. Plant Methods.

[30] Jähne F., Hahn V., Würschum T., Leiser W.L. (2020). Speed breeding short-day crops by LED-controlled light schemes. Theor. Appl. Genet..

[31] Berendsen R.L., Pieterse C.M.J., Bakker P.A.H.M. (2012). The rhizosphere microbiome and plant health. Trends Plant Sci..

[32] Richardson A.E., Barea J.M., McNeill A.M., Prigent-Combaret C. (2009). Acquisition of phosphorus and nitrogen in the rhizosphere and plant growth promotion by microorganisms. Plant Soil.

[33] Nadeem S.M., Ahmad M., Zahir Z.A., Javaid A., Ashraf M. (2014). The role of mycorrhizae and plant growth promoting rhizobacteria (PGPR) in improving crop productivity under stressful environments. Biotechnol. Adv..

[34] Vacheron J., Desbrosses G., Bouffaud M.L., Touraine B., Moënne-Loccoz Y., Muller D., Legendre L., Wisniewski-Dyé F., Prigent-Combaret C. (2013). Plant growth-promoting rhizobacteria and root system functioning. Front. Plant Sci..

[35] Hilou A., Zhang H., Franken P., Hause B. (2014). Do jasmonates play a role in arbuscular mycorrhiza-induced local bioprotection of *Medicago truncatula* against root rot disease caused by *Aphanomyces euteiches*?. Mycorrhiza.

[36] Rybakova D., Cernava T., Köberl M., Liebminger S., Etemadi M., Berg G. (2016). Endophytes-assisted biocontrol: Novel insights in ecology and the mode of action of *Paenibacillus*. Plant Soil.

[37] Huang H.C., Erickson R.S. (2007). Effect of seed treatment with *Rhizobium leguminosarum* on Pythium damping-off, seedling height, root nodulation, root biomass, shoot biomass, and seed yield of pea and lentil. J. Phytopathol..

[38] Faust J.E., Logan J. (2018). Daily light integral: A research review and high-resolution maps of the United States. HortScience.

[39] Fouda A., Eid A.M., Elsaied A., El-Belely E.F., Barghoth M.G., Azab E., Gobouri A.A., Hassan S.E.-D. (2021). Plant Growth-Promoting Endophytic Bacterial Community Inhabiting the Leaves of *Pulicaria incisa* (Lam.) DC Inherent to Arid Regions. Plants.

[40] Fu J., Wang S. (2011). Insights into auxin signaling in plant-pathogen interactions. Front. Plant Sci..

[41] Jiang C., Mithani A., Belfield E.J., Mott R., Hurst L.D., Harberd N.P. (2014). Environmentally responsive genome-wide accumulation of de novo Arabidopsis thaliana mutations and epimutations. Genome Res..

[42] Doudna J.A., Charpentier E. (2014). The new frontier of genome engineering with CRISPR-Cas9. Science.

[43] Hale C.R., Zhao P., Olson S., Duff M.O., Graveley B.R., Wells L., Terns R.M., Terns M.P. (2009). RNA-Guided RNA Cleavage by a CRISPR RNA-Cas Protein Complex. Cell.

[44] Garneau J.E., Dupuis M.È., Villion M., Romero D.A., Barrangou R., Boyaval P., Fremaux C., Horvath P., Magadán A.H., Moineau S. (2010). The CRISPR/cas bacterial immune system cleaves bacteriophage and plasmid DNA. Nature.

[45] Chaudhuri A., Halder K., Datta A. (2022). Classification of CRISPR/Cas system and its application in tomato breeding. Theor. Appl. Genet..

[46] Jackson S.A., McKenzie R.E., Fagerlund R.D., Kieper S.N., Fineran P.C., Brouns S.J.J. (2017). CRISPR-Cas: Adapting to change. Science.

[47] Cao H.X., Wang W., Le H.T.T., Vu G.T.H. (2016). The Power of CRISPR-Cas9-Induced Genome Editing to Speed Up Plant Breeding. Int. J. Genom..

[48] Liu J., Wang S., Wang H., Luo B., Cai Y., Li X., Zhang Y., Wang X. (2021). Rapid generation of tomato male-sterile lines with a marker use for hybrid seed production by CRISPR/Cas9 system. Mol. Breed..

[49] Hamada H., Linghu Q., Nagira Y., Miki R., Taoka N., Imai R. (2017). An in planta biolistic method for stable wheat transformation. Sci. Rep..

[50] Mitter N., Worrall E.A., Robinson K.E., Li P., Jain R.G., Taochy C., Fletcher S.J., Carroll B.J., Lu G.Q., Xu Z.P. (2017). Clay nanosheets for topical delivery of RNAi for sustained protection against plant viruses. Nat. Plants.

[51] Wang M., Lu Y., Botella J.R., Mao Y., Hua K., Zhu J. (2017). Gene Targeting by Homology-Directed Repair in Rice Using a Geminivirus-Based CRISPR/Cas9 System. Mol. Plant.

[52] Fiaz S., Wang X., Younas A., Alharthi B., Riaz A., Ali H. (2021). Apomixis and strategies to induce apomixis to preserve hybrid vigor for multiple generations. GM Crops Food Biotechnol. Agric. Food Chain.

[53] Gaafar R.M., El Shanshoury A.R., El Hisseiwy A.A., AbdAlhak M.A., Omar A.F., Abd El Wahab M.M., Nofal R.S. (2017). Induction of apomixis and fixation of heterosis in Egyptian rice Hybrid1 line using colchicine mutagenesis. Ann. Agric. Sci..

[54] Liu C., He Z., Zhang Y., Hu F., Li M., Liu Q., Huang Y., Wang J., Zhang W., Wang C. (2023). Synthetic apomixis enables stable transgenerational transmission of heterotic phenotypes in hybrid rice. Plant Commun..

[55] Aragão F.J.L., Barros L.M.G., de Sousa M.V., Grossi de Sá M.F., Almeida E.R.P., Gander E.S., Rech E.L. (1999). Expression of a methionine-rich storage albumin from the Brazil nut (*Bertholletia excelsa* H.B.K., Lecythidaceae) in transgenic bean plants (*Phaseolus vulgaris* L., Fabaceae). Genet. Mol. Biol..

[56] Feng Z., Mao Y., Xu N., Zhang B., Wei P., Yang D.-L., Wang Z., Zhang Z., Zheng R., Yang L. (2014). Multigeneration analysis reveals the inheritance, specificity, and patterns of CRISPR/Cas-induced gene modifications in *Arabidopsis*. Proc. Natl. Acad. Sci. USA.

[57] Chen R., Gajendiran K., Wulff B.B.H. (2024). R we there yet? Advances in cloning resistance genes for engineering immunity in crop plants. Curr. Opin. Plant Biol..

[58] Johal G.S., Briggs S.P. (1992). Reductase Activity Encoded by the HM1 Disease Resistance Gene in Maize. Science.

[59] Luo M., Xie L., Chakraborty S., Wang A., Matny O., Jugovich M., Kolmer J.A., Richardson T., Bhatt D., Hoque M. (2021). A five-transgene cassette confers broad-spectrum resistance to a fungal rust pathogen in wheat. Nat. Biotechnol..

[60] Fan X., Jiang H., Meng L., Chen J. (2021). Gene Mapping, Cloning and Association Analysis for Salt Tolerance in Rice. Int. J. Mol. Sci..

[61] Pinho Morais P.P., Akdemir D., Braatz de Andrade L.R., Jannink J., Fritsche-Neto R., Borém A., Couto Alves F., Hottis Lyra D., Granato Í.S.C. (2020). Using public databases for genomic prediction of tropical maize lines. Plant Breed..

[62] Spindel J., Iwata H. (2018). Genomic selection in rice breeding. Rice Genomics, Genetics and Breeding.

[63] Meuwissen T.H.E., Hayes B.J., Goddard M.E. (2001). Prediction of total genetic value using genome-wide dense marker maps. Genetics.

[64] Shamshad M., Sharma A. (2018). The Usage of Genomic Selection Strategy in Plant Breeding. Next Generation Plant Breeding.

[65] Sun J., Khan M., Amir R., Gul A. (2020). Genomic selection in wheat breeding. Climate Change and Food Security with Emphasis on Wheat.

[66] Desta Z.A., Ortiz R. (2014). Genomic selection: Genome-wide prediction in plant improvement. Trends Plant Sci..

[67] Alemu A., Åstrand J., Montesinos-López O.A., Isidro y Sánchez J., Fernández-Gónzalez J., Tadesse W., Vetukuri R.R., Carlsson A.S., Ceplitis A., Crossa J. (2024). Genomic selection in plant breeding: Key factors shaping two decades of progress. Mol. Plant.

[68] Diaz S., Ariza-Suarez D., Ramdeen R., Aparicio J., Arunachalam N., Hernandez C., Diaz H., Ruiz H., Piepho H.P., Raatz B. (2021). Genetic Architecture and Genomic Prediction of Cooking Time in Common Bean (*Phaseolus vulgaris* L.). Front. Plant Sci..

[69] Kumar S. (2020). Epigenomics for Crop Improvement: Current Status and Future Perspectives. J. Genet. Cell Biol..

[70] Schmid M.W., Heichinger C., Coman Schmid D., Guthörl D., Gagliardini V., Bruggmann R., Aluri S., Aquino C., Schmid B., Turnbull L.A. (2018). Contribution of epigenetic variation to adaptation in *Arabidopsis*. Nat. Commun..

[71] Holliday R. (2006). Epigenetics: A Historical Overview. Epigenetics.

[72] Kujur A., Saxena M.S., Bajaj D., Laxmi, Parida S.K. (2013). Integrated genomics and molecular breeding approaches for dissecting the complex quantitative traits in crop plants. J. Biosci..

[73] Köhler C., Springer N. (2017). Plant epigenomics—Deciphering the mechanisms of epigenetic inheritance and plasticity in plants. Genome Biol..

[74] Kawakatsu T., Ecker J.R. (2019). Diversity and dynamics of DNA methylation: Epigenomic resources and tools for crop breeding. Breed. Sci..

[75] Corbin K.R., Bolt B., Rodríguez López C.M. (2020). Breeding for Beneficial Microbial Communities Using Epigenomics. Front. Microbiol..

[76] Das P.R., Sherif S.M. (2020). Application of Exogenous dsRNAs-induced RNAi in Agriculture: Challenges and Triumphs. Front. Plant Sci..

[77] Younis A., Siddique M.I., Kim C.-K., Lim K.-B. (2014). RNA Interference (RNAi) Induced Gene Silencing: A Promising Approach of Hi-Tech Plant Breeding. Int. J. Biol. Sci..

[78] Rajput M., Choudhary K., Kumar M., Vivekanand V., Chawade A., Ortiz R., Pareek N. (2021). RNA Interference and CRISPR/Cas Gene Editing for Crop Improvement: Paradigm Shift towards Sustainable Agriculture. Plants.

[79] Abhary M., Rezk A. (2015). RNAi Technology: A Potential Tool in Plant Breeding. Advances in Plant Breeding Strategies: Breeding, Biotechnology and Molecular Tools.

[80] Chaudhary D., Jeena A.S., Rohit, Gaur S., Raj R., Mishra S., Kajal, Gupta O.P., Meena M.R. (2024). Advances in RNA Interference for Plant Functional Genomics: Unveiling Traits, Mechanisms, and Future Directions. Appl. Biochem. Biotechnol..

[81] Enrique R., Siciliano F., Favaro M.A., Gerhardt N., Roeschlin R., Rigano L., Sendin L., Castagnaro A., Vojnov A., Marano M.R. (2011). Novel demonstration of RNAi in citrus reveals importance of citrus callose synthase in defence against *Xanthomonas citri* subsp. *citri*. Plant Biotechnol. J..

[82] Jiang C.-J., Shimono M., Maeda S., Inoue H., Mori M., Hasegawa M., Sugano S., Takatsuji H. (2009). Suppression of the Rice Fatty-Acid Desaturase Gene OsSSI2 Enhances Resistance to Blast and Leaf Blight Diseases in Rice. Mol. Plant-Microbe Interact..

[83] Baum J.A., Bogaert T., Clinton W., Heck G.R., Feldmann P., Ilagan O., Johnson S., Plaetinck G., Munyikwa T., Pleau M. (2007). Control of coleopteran insect pests through RNA interference. Nat. Biotechnol..

[84] Manavalan L.P., Chen X., Clarke J., Salmeron J., Nguyen H.T. (2012). RNAi-mediated disruption of squalene synthase improves drought tolerance and yield in rice. J. Exp. Bot..

[85] Wang J., Zhang L., Cao Y., Qi C., Li S., Liu L., Wang G., Mao A., Ren S., Guo Y.-D. (2018). CsATAF1 Positively Regulates Drought Stress Tolerance by an ABA-Dependent Pathway and by Promoting ROS Scavenging in Cucumber. Plant Cell Physiol..

[86] Guan Q., Lu X., Zeng H., Zhang Y., Zhu J. (2013). Heat stress induction of mi R 398 triggers a regulatory loop that is critical for thermotolerance in Arabidopsis. Plant J..

[87] Wu J., Yu C., Huang L., Gan Y. (2021). A rice transcription factor, *OsMADS57*, positively regulates high salinity tolerance in transgenic *Arabidopsis thaliana* and *Oryza sativa* plants. Physiol. Plant..

[88] Rosa C., Kuo Y.-W., Wuriyanghan H., Falk B.W. (2018). RNA Interference Mechanisms and Applications in Plant Pathology. Annu. Rev. Phytopathol..

[89] Ketumile D., Yang X., Sanchez R., Kundariya H., Rajewski J., Dweikat I.M., Mackenzie S.A. (2022). Implementation of Epigenetic Variation in Sorghum Selection and Implications for Crop Resilience Breeding. Front. Plant Sci..

[90] Tonosaki K., Fujimoto R., Dennis E.S., Raboy V., Osabe K. (2022). Will epigenetics be a key player in crop breeding?. Front. Plant Sci..

[91] Jiang F., Doudna J.A. (2017). CRISPR–Cas9 Structures and Mechanisms. Annu. Rev. Biophys..

[92] Zheng X., Chen L., Xia H., Wei H., Lou Q., Li M., Li T., Luo L. (2017). Transgenerational epimutations induced by multi-generation drought imposition mediate rice plant’s adaptation to drought condition. Sci. Rep..

[93] Ahmar S., Gill R.A., Jung K.H., Faheem A., Qasim M.U., Mubeen M., Zhou W. (2020). Conventional and Molecular Techniques from Simple Breeding to Speed Breeding in Crop Plants: Recent Advances and Future Outlook. Int. J. Mol. Sci..

[94] Teressa T., Semagehn Z., Bejiga T. (2021). Multi Environments and Genetic-Environmental Interaction (GxE) in Plant Breeding and its Challenges: A Review Article. Int. J. Res. Stud. Agric. Sci..

[95] Hudson A.I., Odell S.G., Dubreuil P., Tixier M.-H., Praud S., Runcie D.E., Ross-Ibarra J. (2022). Analysis of genotype-by-environment interactions in a maize mapping population. G3 Genes Genomes Genet..

[96] Egea-Gilabert C., Pagnotta M.A., Tripodi P. (2021). Genotype × Environment Interactions in Crop Breeding. Agronomy.

[97] Budhlakoti N., Kushwaha A.K., Rai A., Chaturvedi K.K., Kumar A., Pradhan A.K., Kumar U., Kumar R.R., Juliana P., Mishra D.C. (2022). Genomic Selection: A Tool for Accelerating the Efficiency of Molecular Breeding for Development of Climate-Resilient Crops. Front. Genet..

[98] Jannink J.-L., Lorenz A.J., Iwata H. (2010). Genomic selection in plant breeding: From theory to practice. Brief. Funct. Genom..

[99] Saini H., Thakur R., Gill R., Tyagi K., Goswami M. (2023). CRISPR/Cas9-gene editing approaches in plant breeding. GM Crops Food Biotechnol. Agric. Food Chain.

[100] Halder K., Chaudhuri A., Abdin M.Z., Majee M., Datta A. (2022). RNA Interference for Improving Disease Resistance in Plants and Its Relevance in This Clustered Regularly Interspaced Short Palindromic Repeats-Dominated Era in Terms of dsRNA-Based Biopesticides. Front. Plant Sci..

[101] Singh I., Sheoran S., Kumar B., Kumar K., Rakshit S. (2021). Speed breeding in maize (Zea mays) vis-à -vis in other crops: Status and prospects. Indian J. Agric. Sci..

[102] Farooqi M.Q.U., Nawaz G., Wani S.H., Choudhary J.R., Rana M., Sah R.P., Afzal M., Zahra Z., Ganie S.A., Razzaq A. (2022). Recent developments in multi-omics and breeding strategies for abiotic stress tolerance in maize (*Zea mays* L.). Front. Plant Sci..

[103] Butrón A., Santiago R., Gowda M. (2023). Editorial: Model organisms in plant science: Maize. Front. Plant Sci..

[104] Hake S., Ross-Ibarra J. (2015). Genetic, evolutionary and plant breeding insights from the domestication of maize. eLife.

